# IL-17RA–Mediated Epithelial Cell Activity Prevents Severe Inflammatory Response to *Helicobacter pylori* Infection

**DOI:** 10.4049/immunohorizons.2300078

**Published:** 2024-04-19

**Authors:** Lee C. Brackman, Matthew S. Jung, Eseoghene I. Ogaga, Nikhita Joshi, Lydia E. Wroblewski, M. Blanca Piazuelo, Richard M. Peek, Yash A. Choksi, Holly M. Scott Algood

**Affiliations:** * Tennessee Valley Healthcare System, Department of Veterans Affairs, Nashville, TN; † Division of Infectious Disease, Department of Medicine, Vanderbilt University School of Medicine, Vanderbilt University Medical Center, Nashville, TN; ‡ Vanderbilt University, School of Biological Sciences, Nashville, TN; § Division of Gastroenterology, Department of Medicine, Vanderbilt University School of Medicine, Vanderbilt University Medical Center, Nashville, TN; ¶ Department of Pathology, Microbiology and Immunology, Vanderbilt University School of Medicine, Vanderbilt University Medical Center, Nashville, TN; ǁ Vanderbilt Institute of Infection, Immunity, and Inflammation, Vanderbilt University Medical Center, Nashville, TN

## Abstract

*Helicobacter pylori* is a Gram-negative pathogen that colonizes the stomach, induces inflammation, and drives pathological changes in the stomach tissue, including gastric cancer. As the principal cytokine produced by Th17 cells, IL-17 mediates protective immunity against pathogens by inducing the activation and mobilization of neutrophils. Whereas IL-17A is largely produced by lymphocytes, the IL-17 receptor is expressed in epithelial cells, fibroblasts, and hematopoietic cells. Loss of the IL-17RA in mice results in impaired antimicrobial responses to extracellular bacteria. In the context of *H. pylori* infection, this is compounded by extensive inflammation in *Il17ra^−/−^* mice. In this study, *Foxa3^cre^Il17ra^fl/fl^* (*Il17ra^ΔGI-Epi^*) and *Il17ra^fl/fl^* (control) mice were used to test the hypothesis that IL-17RA signaling, specifically in epithelial cells, protects against severe inflammation after *H. pylori* infection. The data indicate that *Il17ra^ΔGI-Epi^* mice develop increased inflammation compared with controls. Despite reduced *Pigr* expression, levels of IgA increased in the gastric wash, suggesting significant increase in Ag-specific activation of the T follicular helper/B cell axis. Gene expression analysis of stomach tissues indicate that both acute and chronic responses are significantly increased in *Il17ra^ΔGI-Epi^* mice compared with controls. These data suggest that a deficiency of IL-17RA in epithelial cells is sufficient to drive chronic inflammation and hyperactivation of the Th17/T follicular helper/B cell axis but is not required for recruitment of polymorphonuclear neutrophils. Furthermore, the data suggest that fibroblasts can produce chemokines in response to IL-17 and may contribute to *H. pylori*–induced inflammation through this pathway.

## Introduction

*Helicobacter pylori* is a Gram-negative pathogen that chronically colonizes the human gastric epithelium and can cause severe disease outcomes such as gastric cancer (1, 2). Although *H. pylori* infection is recognized as the primary risk factor for gastric cancer (3), it is not the only infectious agent that can drive cancer (4). Carcinogenesis associated with infectious processes is often impacted by the inflammatory processes activated by pathogens (5). During *H. pylori* infection, more gastric inflammation (termed gastritis) is associated with worse pathological consequences, including atrophic gastritis, dysplasia, and cancer (6–8).

The chronic inflammatory processes activated in response to *H. pylori* infection are primarily mediated by CD4^+^ Th cell activities (9, 10). Depending on the cytokine environment when MHC(Ag):TCR activation occurs, naive CD4^+^ T cells can be induced to express different cytokines and differentiate to specific subsets of Th cells (11). The balance between these subsets impacts the extent of the pathological outcomes during *H. pylori* infection (9). For instance, an increase in T regulatory cells (Tregs) results in a reduced Th1/Treg or Th17/Treg ratio, subsequently mitigating inflammation and disease progression, in contrast to scenarios characterized by heightened Th1 and Th17 responses (12–14). A study out of South Korea on *H. pylori*–positive and *H. pylori*–negative children with dyspeptic symptoms found that FOXP3-expressing Tregs and an associated increase in TGF-β1 expression were significantly increased in *H. pylori*–positive children compared with *H. pylori*–negative children, and this correlated positively with *H. pylori* density (12). *H. pylori*–infected children in Santiago, Chile had reduced levels of gastric inflammation (including reduced polymorphonuclear neutrophil [PMN] accumulation) compared with adults, which correlated with reduced Th17 cells and reduced IL-17A production, but increased IL-10 levels (14). Assessing ratios of Th17/Tregs by immunohistochemistry and quantitative PCR (qPCR) in tumor samples indicated that accumulation of Th17 and Tregs in the tumor microenvironment was gradually increased according to disease progression (13). The authors suggest that this is evidence that an imbalance in Th17/Tregs is involved in development and progression of disease. In fact, the development of tolerance, which occurs more often in animal models infected at an earlier age, protected mice from gastric cancer precursor lesions (15).

The Th1 and Th17 responses in the gastric mucosa are also linked to inflammation driven by their signature cytokines, which communicate with and activate other mononuclear cells. For example, Th1 responses result in the production of IFN-γ, and IFN-γ impacts *H. pylori*–driven inflammation in mouse models (16, 17). IFN-γ activates Ag presentation pathways (18, 19), recruitment of macrophages, and production of reactive oxygen and nitrogen species (20). Th17 responses result in the production of IL-17a, IL-17f, IL-21, and IL-22, have been shown to activate many cell types, and drive increased expression of neutrophil-recruiting chemokines as well as antimicrobial proteins (21, 22). Neutrophil infiltration can play a pivotal role in the management of bacterial burden in many extracellular infection responses (23); however, it may concurrently induce tissue damage (24). The inflammatory response associated with neutrophil infiltration is also associated with carcinogenesis and metastasis (reviewed in Refs. 25, 26).

In the context of *H. pylori*, an extracellular pathogen that drives chronic inflammation and contributes to gastric cancer, the role of the Th17 response is complex and somewhat controversial. Several studies conclude that increases in IL-17A production correlate with severe disease and poor prognosis (27–30). For example, there were increased Th17 cells in tumors of patients with advanced gastric cancer, and hence the data suggest that IL-17 may enhance disease progression (27–29). Another study found elevated IL-17A expression in lymph nodes of patients with lymph node metastasis compared with those without metastasis (30). Additionally, the same study reported that higher IL-17A expression was observed in the tumors from patients with advanced gastric cancer compared with early cancer (30). Mouse models have allowed the role of IL-17 to be investigated in a controlled setting. It is clear from these studies that Th17 responses, especially IL-17A, have been shown to play an important role in the inflammatory response during *H. pylori* colonization of the gastric mucosa (31) and is clearly important for recruitment of PMNs during *H. pylori* infections (32, 33). A somewhat unexpected finding from our mouse infection model was that the *H. pylori*–infected *Il17ra*^−/−^ mice, which lack a functional IL-17 receptor, have higher levels of chronic inflammation than do *H. pylori*–infected control mice independent of the background of the mice (C57BL/6 or FVB/N) (32, 34). This was evident also in the development of secondary lymphoid compartments in the gastric tissue (32, 34).

It was previously demonstrated that human gastric epithelial cell lines respond to IL-17 in vitro to produce some antimicrobials and chemokines especially when a costimulus such as IL-22 or TNF is provided (33). Furthermore, these stimuli, IL-22 and IL-17a, can induce antimicrobials that can inhibit the growth of *H. pylori* (35). One subunit of the IL-17 receptor, IL-17RA, is expressed on many cell types and not only responds to IL-17A, but also to other family members, including IL-17F and IL-17A/F (36). Despite the broad expression of the IL-17 receptor across various cell types, the research emphasis during *H. pylori* infection has mostly centered on exploring IL-17 expression and correlations with disease outcomes, and a handful of studies explore IL-17a activation of gastric epithelial cells (35, 37, 38). It is very clear that the IL-17RA molecule is responsible for modulating inflammation in vivo, as IL-17RA–deficient mice have very high inflammatory responses after *H. pylori* infection (32, 34), but how specific cells contribute to these pathways is not clear. Interestingly, experiments with *Il17a^−/−^* mice do not develop the same phenotypes as the *Il17ra*^−/−^ mice, suggesting that there is potentially compensation through IL-17F (33). In previous studies, we explored the hypothesis that the absence of IL-17RA could lead to the loss of a negative feedback loop in T cells potentially accounting for the exacerbated expression of IL-17A and IL-21 in *H. pylori*–infected *Il17ra^−/−^* mice (33). Using the *Cd4^cre^Il17ra^fl/fl^* model, the data indicated that the loss of IL-17RA signaling in T cells was not sufficient to recapitulate the hyperinflammatory response observed with the loss of IL-17RA (*Il17ra^−/−^* mice) (33). In this study, the goal was to determine the epithelial cell–specific response to IL-17RA signaling and the contribution of IL-17 signaling in epithelial cells to modulation of the proinflammatory *H. pylori*–specific Th/B cell responses. The data presented in this study suggest that IL-17 receptor signaling in epithelial cells plays a critical role in maintaining a proper inflammatory response toward *H. pylori.* Furthermore, the findings suggest that fibroblasts may facilitate neutrophil recruitment through their responsiveness to IL-17.

## Materials and Methods

### Ethics statement

This study was accomplished under protocol no. V2000068 and was approved by Institutional Animal Care and Use Committee of Vanderbilt University Medical Center and the Research and Development Committee of the Veterans Affairs Tennessee Valley Healthcare System. Experiments were executed in accordance with American Association for the Accreditation of Laboratory Animal Care guidelines, the American Veterinary Medical Association *Guidelines for the Euthanasia of Animals*, National Institutes of Health regulations (*Guide for the Care and Use of Laboratory Animals*), and the United States Animal Welfare Act. All animals were housed in an accredited research animal facility that is fully staffed with trained personnel.

### Animals

Breeders for each experimental group were acquired from multiple sources for this project. The original breeding pairs of *Il17ra^fl/fl^* mice were acquired through a material transfer agreement with Michael Karin (University of California, San Diego). *Foxa3*^Cre^ transgenic mice were obtained from Keith Wilson (originally created by Klaus Kaestner [39], (Tg(Foxa3-cre)1Khk transgene detail MGI mouse; https://www.informatics.jax.org/allele/MGI:2664968). *Vil1^Cre^* transgenic mice were obtained from The Jackson Laboratory (Bar Harbor, ME; strain 021504). The experimental groups and littermate controls were generated by mating *Foxa3^cre^Il17ra^fl/fl^* mice with *Il17ra^fl/fl^* mice or by mating *Vil1^cre^Il17ra^fl/fl^* mice with *Il17ra^fl/fl^* mice. *Il17ra^fl/fl^* mice were identified by a 572-bp band, and *Foxa3^cre^Il17ra^fl/f^* mice were identified by a 250-bp band using the following primer sets:

**Table:** 

Genotype	Primer set 1	Primer set 2	Primer set 3
Foxa3^cre^	GCGGCATGGTGCAAGTTGAAT	CGTTCACCGGCATCAACGTTT	—
Il17ra^fl/fl^	GGGGTTTTTGTTGTTGTTGG	GCAGCTGTTCTCAACCTTCC	GGCCAGGATCTACCACAAAG

Cre-positive animals were identified through PCR of tail snip lysate followed by gel electrophoresis in-house, and later through qPCR (Transnetyx, Memphis, TN). C56BL/6 breeders that were compared with germline *Il17ra^−/−^* (Amgen, Material Transfer Agreement) were obtained from Taconic Biosciences and have been breeding at the Vanderbilt University School of Medicine for generations. Feces from sentinel mice in the same housing facility tested negative for *H. pylori*, pinworms, mouse parvovirus, and other murine pathogens.

### Establishing efficacy and specificity of Foxa3-Cre in gastric tissue

*Foxa3^cre^* mice have been used to establish gastrointestinal epithelial cell conditional knockout mice (40–44). To address efficiency in gastric epithelial cells, a gastric epithelial cell isolation protocol was adapted (45, 46). Stomachs were extracted from mice, and forestomaches were removed and sectioned into three to four pieces. Gastric tissue pieces were then incubated on ice in 10 ml of 0.5 mM DTT/0.5 M EDTA. After 30 min, EDTA/DTT solution was discarded and replaced with 10 ml of 3 mM EDTA solution. After another 10-min incubation, tubes were shaken vigorously for 1 min. The supernatant was collected, and the process was repeated three to five times with 3m M EDTA. Afterwards, the cell suspension was poured through a 70-µm cell strainer and centrifuged at 433 × *g* for 10 min at 4°C. The supernatant was removed and TRIzol was added for RNA isolate (see below for protocol).

Gastric epithelial cells isolated from *Foxa3^cre^Il17ra^fl/fl^* mice were deficient for *Il17ra* expression (Fig. 3), but expression was retained in *Il17ra^fl/fl^* mice. Furthermore, expression of *Il17ra* was retained in gastric fibroblasts sorted from stomachs of *Foxa3^cre^Il17ra^fl/fl^* mice similar to levels in control mice (Fig. 3). A bioassay was also performed on gastric fibroblast cultures from *Foxa3^cre^Il17ra^fl/fl^* mice compared with germline *Il17ra^−/−^* and C57BL/6 fibroblasts (see *Primary fibroblast cultures* and Fig. 8) It is noteworthy that endothelial cells do not express *Il17ra* at levels above background in any genotype tested. For the purposes of this study, we use this nomenclature to describe the conditional knockout, *Il17ra^ΔGI-epi^*.

### Gastric organoid isolation and monolayer conversion

The protocol for isolation of gastric organoids (henceforth referred to as gastroids) was adapted from Bartfeld and Clevers (47). Stomachs were dissected out and forestomaches were removed from 8- to 12-wk-old C57BL/6 mice. Tissue was sliced into 1-mm pieces, placed in 5 mM EDTA, and rocked in a cold room for 30 min. EDTA was removed and tissue was washed in 5 ml of Dulbecco’s PBS, after which glands were isolated with a glass slide and centrifuged at 300 × *g* for 10 min at 4°C before being resuspended in Matrigel (356237, BD Biosciences, Franklin Lakes, NJ). Then, 50 µl of the resuspension was then plated in a 12-well tissue culture–treated plate and placed in a 37°C incubator for 15 min to allow Matrigel to fully polymerize. After 15 min the Matrigel was overlaid with 50% conditioned media from L-WRN cells (48).

Gastroids were maintained by changing media every 2 d and passaging every 4–7 d as needed. Gastroids were converted to monolayers by pipetting organoids to shear and disrupt the Matrigel. Sheared organoids were centrifuged at 720 × *g* for 5 min at 4°C, incubated in 1 ml of 0.05% trypsin/EDTA for 10 min at 37°C before 10 ml of medium containing FBS was added to neutralize the reaction. Single cells were resuspended in cell growth media and plated in tissue culture plates or transwells.

After 24–48 h, when monolayers were 80–90% confluent by visualization, the cells were stimulated with recombinant murine IL-17a, IL-17f, or IL-17a/f (R&D Systems) at 100 ng/ml for 12 h. RNA isolation was performed using a Qiagen microRNA kit per the manufacturer’s instructions and real-time qPCR was performed.

### Bacterial strain and growth conditions and mouse infection

All assays were conducted using *H. pylori* premouse Sydney strain 1 (PMSS1), which retains the type 4 secretion system, a key oncogenic factor of *H. pylori.* Bacteria cultures were grown on trypticase soy agar plates containing 5% sheep blood (Thermo Fisher Scientific, Waltham, MA) and incubated at 37°C in 5% CO_2_ and passaged every 48 h. To grow the mouse inoculum, the bacteria were grown in liquid culture (*Brucella* broth with 10% heat-inactivated FBS and 10 μg/ml vancomycin) at 37°C while shaking at 160 rpm for 16–18 h under microaerophilic conditions generated by a GasPak EZ CampyPac container system (Becton Dickinson, Franklin Lakes, NJ). Bacterial concentration was verified via OD_600_ on the BioTek ELx808 plate reader and Gen5 3.10 software (BioTek, Winooski, VT).

At 8–10 wk of age, mice were inoculated by oral gavage with two doses of *H. pylori* PMSS1 (1 × 10^9^ CFU/ml) in 0.5 ml of *Brucella* broth while under sedation from 3–5% isoflurane (VetEquip, Pleasanton, CA). These doses were given ∼48 h apart. The mice were euthanized at 1, 2, or 3 mo postinfection and tissue was collected for subsequent analysis.

### Harvest and processing of stomach

The stomach was removed after opening the peritoneal cavity and making an excision at the distal esophagus and duodenum. Once removed, a cross-sectional incision separated the forestomach (nonglandular) from the glandular stomach (antrum and corpus). The nonglandular portion of the stomach was discarded. The glandular stomach was then opened by an incision along the lesser curvature and added to 1.0 ml of cold PBS with protease inhibitor (cOmplete tablets, EASYpack protease inhibitor cocktail tablets; Roche, Basel, Switzerland) and shaken vigorously for 15 s. The tissue was then removed and sectioned, which is detailed below. The PBS and protease inhibitor mixture (henceforth referred to as gastric wash) was stored on ice until the end of the harvest and then centrifuged at 3500 × *g* for 10 min at 4°C. The supernatant was then removed and stored at −80°C for downstream analyses. The stomach was cut into three strips lengthwise to capture the duodenum, pylorus, antrum, corpus, and squamocolumnar junction. The first strip was added to 10% normal buffered formalin for 24 h, embedded in paraffin, and stained with H&E to examine histological changes. The second strip was frozen at −80°C for subsequent gene expression analysis. The third strip was added to a preweighed tube containing 600 µl of *Brucella* broth with 10% FBS, weighed, and homogenized in the Fisherbrand Bead Mill 24 (Thermo Fisher Scientific). Samples were then normalized to 1.0 ml and plated at dilutions of 10^2^, 10^3^, and 10^4^ on trypticase soy agar plates containing 5% sheep blood with nalidixic acid (10 µg/ml), vancomycin (50 µg/ml), amphotericin (2 µg/ml), and bacitracin (100 µg/ml). Plates were stored in airtight containers with BD GasPak EZ sachets (260680, Becton Dickinson) at 37°C for 6 d. CFU were counted, normalized to grams of stomach tissue homogenized, and log transformed to determine differences in bacterial burden between genotypes.

### Histological scoring

Inflammation was scored by a single pathologist, who was blinded to the experimental versus control groups at the time of scoring. Using the updated Sydney system, both acute and chronic inflammation were graded on a scale from 0 to 3 as follows: no inflammation (grade 0), mild inflammation (grade 1), moderate inflammation (grade 2), and severe inflammation (grade 3). Acute inflammation scores were based on the density of neutrophils, and chronic inflammation scores were based on the density of lamina propria mononuclear cell infiltration (mainly lymphocytes, plasma cells, and macrophages) within each sample. Total inflammation was determined as the sum of acute and chronic inflammation within the corpus or the antrum, resulting in inflammation scores on a scale from 0 to 12. Lymphoid follicles and aggregates were recorded as counts in the entire length of the stomach for each mouse. The numbers of follicles and aggregates are combined to report the number of lymphoid follicles per section, the axis reads lymphoid follicles (number of aggregates/section).

### Quantitative multiplex nucleic acid hybridization assay and analysis

Multiplex nucleic acid hybridization technology was used to quantify gene expression. For this assay, RNA was isolated from tissue using TRIzol reagent as described in previous work (33). RNA quality and quantity were assessed both by a NanoDrop spectrophotometer and through the VANTAGE Core at Vanderbilt University Medical Center with a Bioanalyzer (Agilent Technologies). For the multiplex nucleic acid hybridization assay (NanoString Technologies, Seattle, WA) input RNA was hybridized at 65°C overnight to an nCounter NanoString mouse immunology panel that contained >550 barcoded reporter probes (code set: XT-CSO-MIM1-12). These hybridized samples were loaded onto an nCounter SPRINT Cartridge, and gene transcript abundance was determined using the nCounter SPRINT Profiler (NanoString Technologies). The nCounter NanoString mouse immunology panel measured the expression of 561 target genes including up to 15 internal control genes. Data were analyzed by Rosalind (https://rosalind.bio/; version 3.16, 2023), with a HyperScale architecture developed by Rosalind (San Diego, CA). Violin plots were generated as part of the quality control step. Normalization, fold changes, and *p* values were calculated using criteria provided by NanoString. Rosalind follows the nCounter advanced analysis protocol of dividing counts within a lane by the geometric mean of the normalizer probes from the same lane. Housekeeping probes to be used for normalization are selected based on the geNorm algorithm as implemented in the NormqPCR. Fold changes and *p* values were calculated within the software using the fast method as described in the nCounter advanced analysis 2.0 user manual. The *p* value adjustment is performed using the Benjamini–Hochberg method of estimating false discovery rates. Rosalind referenced several database sources for enrichment analysis, including InterPro, National Center for Biotechnology Information, MSigDB, Reactome, and WikiPathways. Enrichment was calculated relative to a set of background genes relevant for the experiment.

### Quantitative real-time PCR

RNA was isolated from tissue and cell samples using TRIzol reagent as directed by the manufacturer. cDNA was then reverse transcribed using a high-capacity cDNA reverse transcription kit (Applied Biosystems, Foster City, CA) and diluted for real-time PCR. A StepOnePlus PCR machine (Applied Biosystems) was used to run TaqMan (Thermo Fisher Scientific)-based gene expression assays, relative to uninfected or unstimulated controls and normalized to *Gapdh* expression. Data are reported as relative units (RU), which utilize the formula RU = 2^−ΔΔCt^ to normalize the levels of expression to both the internal housekeeping gene and a calibration sample (uninfected tissue or unstimulated cells).

Murine TaqMan gene expression assays included the following: *Gapdh* (Mm99999915_g1), *Il17a* (Mm00439619_m1), *Il21* (Mm00517640_m1), *Cd19* (Mm00515420_m1), *Pigr* (Mm00465049_m1) *Nox1* (Mm00549170_m1), *Cxcl1* (Mm04207460_m1), *Cxcl2* (Mm00436450_m1), *Cxcl5* (Mm00436451_g1), *S100a8* (Mm00496696_g1), *S100a9* (Mm00656925_m1), *Epcam* (Mm00493214_m1), and *Il17ra* (Mm00493214_m1).

### ELISAs for IgA levels

Total IgA levels were measured in gastric wash samples using the Invitrogen IgA mouse uncoated ELISA kit (88-05450-22, Thermo Fisher Scientific) following the manufacturer’s instructions. Dilutions of gastric wash (1:5, 1:25, and 1:125) were prepared in assay buffer (1× PBS, 0.05% Tween 20, and 0.5% BSA) for each sample. Total IgA concentration was analyzed relative to a second-order polynomial standard curve.

To determine *H. pylori*-specific Ab, 96-well plates were coated with 100 μl of 10 μg/ml *H. pylori* PMSS1 lysate overnight at 4°C. After washing with wash buffer (00-0400-59, Thermo Fisher Scientific), wells were blocked with blocking buffer (1× PBS, 1% Tween 20, and 10% BSA) for 2 h at room temperature before washing again. Samples were added at 1:5, 1:25, and 1:125 dilution in 1× PBS before incubating overnight at 4°C. After washing, 100 μl/well goat anti-mouse IgA-HRP (1040-05, SouthernBiotech, Birmingham, AL) diluted 1:6000 was added and incubated for 1 h before washing again. Color was developed by incubation with tetramethylbenzidine substrate (88-50450-88, Thermo Fisher Scientific) and then stopped after 15 min with the addition of 1 M H_2_SO_4_. The plate was then immediately read at 450 nm (BioTek ELx808). Data were reported as fold change above the OD reading of gastric wash samples from *H. pylori–*negative mice.

### Primary fibroblast cultures

Fibroblasts were extracted from the stomachs of C57BL/6, *Il17ra^ΔGI-epi^*, and *Il17ra^−/−^* C57BL/6 mice. In short, 5 mM EDTA prepared in chelating buffer (54.9 mM d-sorbitol and 43.4 mM sucrose in Dulbecco’s PBS) was used to extract and discard gastric epithelial cells. Furthermore, to extract the fibroblasts, the gastric tissue was digested with 100 µg/ml collagenase (17018-029, Life Technologies) and 1 µg/ml Dispase (54905400, Roche) for 2 h while vortexing vigorously every 20 min. The cell suspension was then poured over a 70-µm cell strainer and centrifuged at 400 × *g* for 5 min at 4°C. Primary fibroblasts were resuspended and cultured in advanced DMEM F12 (12634010; Life Technologies) containing 10% FBS, 1% penicillin/streptomycin, and 0.2% Primocin for up to six passages. For stimulations, cells were serum starved overnight and then stimulated with 20–100 ng/ml rIL-17a (210-17, PeproTech, Rocky Hill, NJ) or 0.1% BSA in PBS for 6 h. RNA was isolated as described above, and real-time qPCR was performed using TaqMan gene expression assays for *Cxcl1* (Mm04207460_m1), *Cxcl2* (Mm00436450_m1), *Cxcl5* (Mm00436451_g1), and *Il17ra* (Mm00493214_m1) (Applied Biosystems/Thermo Fisher Scientific).

### Statistical analysis

Statistical significance is based on a one-way ANOVA test with a Dunnett multiple comparison test for cellular stimulation assays with multiple cytokines, an unpaired *t* test assuming Gaussian distribution for real-time qPCR analyses, log-transformed CFU/ml values, and Ab levels. For inflammation scoring, statistical significance was determined after running a Mann–Whitney *U* test. These analyses were run using GraphPad Prism 9.5.0 software (GraphPad, Boston, MA). Unless noted differently, individual data points are shown with a line representing the mean ± SEM. Where relevant, statistical significance is marked as follows: *p < 0.05, **p < 0.01, ***p < 0.001, *** *p* < 0.0001.

## Results

### Higher expression of the IL-17RA gene is a favorable prognostic marker for stomach cancer survival

**FIGURE 1.**
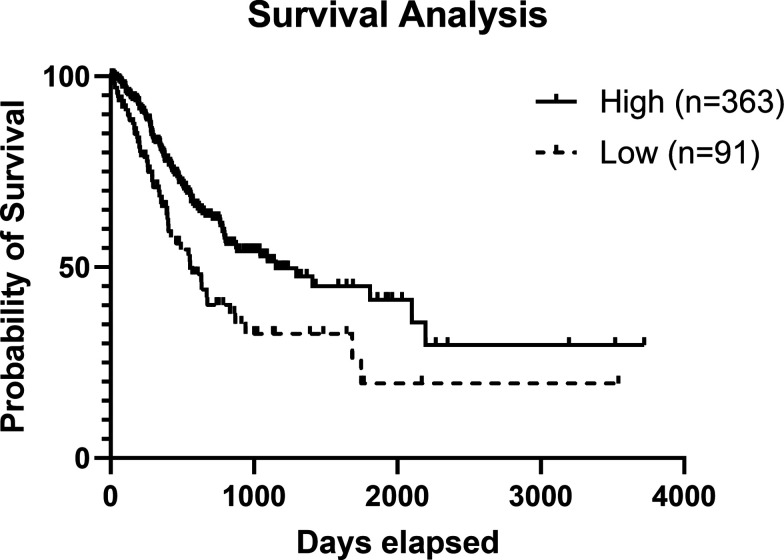
Higher expression of the IL-17RA gene is a favorable prognostic marker for stomach cancer survival. Kaplan–Meier plots summarize results from analysis of the correlation between mRNA expression level and patient survival (*p* = 0.0010). Patients were divided based on level of expression into one of the two groups: low (under cutoff, *n* = 91) or high (over cutoff, *n* = 363). The cutoff used for this analysis was based on the best predictable FPKM (fragments per kilobase of transcript per million mapped reads) value using the Human Protein Atlas tool (currently set at 3.8). Data from the Human Protein Atlas was extracted on September 15, 2023 to generate this Kaplan–Meier plot. The *x*-axis shows time for survival (days) and the *y*-axis shows the probability of survival as a percent.

Several years ago, Human Protein Atlas program data were made publicly available (49–52). The database was made fully accessible to allow for exploration of the human proteins in cells, tissues, and organs. The Pathology Section of the Human Protein Atlas (53) allows users to examine the impact of high or low protein levels on survival of patients with cancer. Therefore, this database can provide favorable or unfavorable prognostic markers for cancer survival. By utilizing the open-access tool from the Human Protein Atlas and investigating whether expression of IL-17RA has any prognostic value, it is evident that IL-17RA expression is a favorable prognostic marker for stomach cancer survival (*p* < 0.001, Fig. 1). This finding provides evidence that the IL-17RA pathway is relevant to human disease progression. Although it was previously demonstrated that the deficiency in IL-17RA in mice resulted in elevated levels of inflammation, this finding in humans has prompted further study.

### IL-17 signaling in epithelial cells regulates several antimicrobial factors and chemokines

**FIGURE 2.**
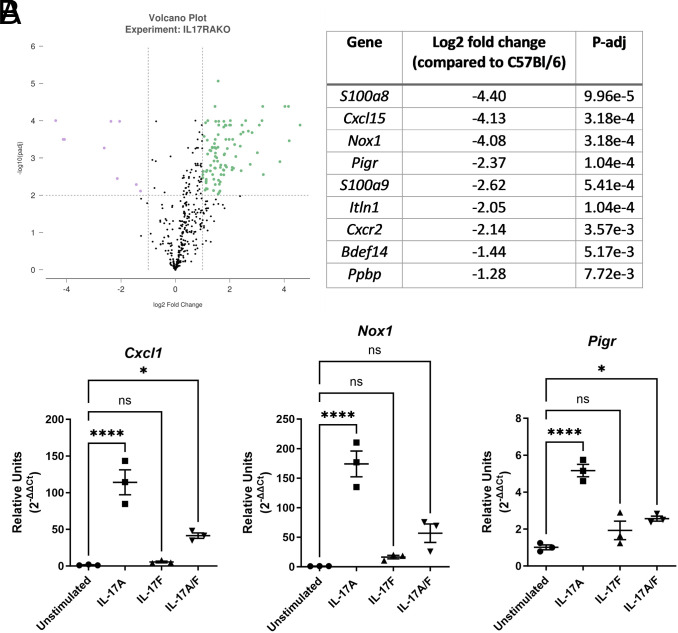
IL-17 signaling impacts expression of genes that contribute to innate barrier function and restricts chronic inflammation. (**A**) Differential abundance of genes in the stomach at 3 mo after *H. pylori* infection in *Il17ra^−/−^* mice compared with C57BL/6 mice using the nCounter Immunology NanoString panel. The table represents genes that are lower in expression in *Il17ra^−/−^* with a log_10_ adjusted *p* value of >2 and a log_2_ fold change of greater than −1.25. On the volcano plot, these genes are in purple. The multiplex RNA hybridization assay was performed on six mice per genotype. (**B**) Gastric murine organoids from C57BL/6 mice (gastroids) were stimulated with 50 ng/ml IL-17A, IL-17F, and the heterodimer IL-17A/F. RNA was then extracted from these gastroids and qPCR assays of genes associated with epithelial cell responses including *Cxcl1*, *Nox1*, and *Pigr* were measured. *Gapdh* was used as an endogenous control, and unstimulated gastroids were pooled and used as a reference sample. The data are representative of three independent experiments. Error bars represent ± SEM. One-way ANOVA with a Dunnett multiple comparison test was used to determine significance. *p < 0.05, ****p < 0.0001, compared with unstimulated.

The importance of IL-17 signaling through the IL-17RA molecule has been investigated in many models of extracellular bacterial infection (54–58). *H. pylori* infection of *Il17ra^−/−^* mice was performed in knockout mice both on the C57BL/6 and FVB/N backgrounds, and the data indicate that IL-17RA is required to control chronic inflammation (32–34). To understand further impact of the mutation on inflammation, a multiplex RNA hybridization assay was performed at 3 mo postinfection and the differential gene expression of a panel of >500 genes was assessed. The data indicate that, in *H. pylori*–infected *Il17ra^−/−^*mice, several genes encoding antimicrobial proteins or encoding proteins associated with neutrophils exhibit reduced abundance compared with *H. pylori*–infected wild-type mice (Fig. 2A), including *S100a8*, *S100a9*, *Bdef14*, *Cxcr2*, *Nox1*, and *Pigr*. Genes that are upregulated are largely genes associated with chronic inflammation and adaptive immune responses (Supplemental Table I). Among some of the most significantly upregulated genes are *Cd19* (a B cell marker), *Cxcr5* and *Il21r* (genes associated with T follicular helper cells), *H2* genes (MHC class II genes), and *Il17a.* It is noteworthy that *Il17ra* is on the list of genes with increased transcript counts in the *Il17ra^−/−^* mice (log_2_ fold change = 1.57, adjusted *p* = 8.64E-06). This can be explained by the fact that the hybridization probe used in the NanoString panel corresponds to a 100-bp sequence (nt 325–424) upstream of the deletion (corresponding to nt 445–1172).

Because many of the genes downregulated in the *Il17ra^−/−^* mice are also associated with epithelial cell responses, the ability of recombinant IL-17 proteins including IL-17A, IL-17F, and IL-17A/F to activate primary epithelial cells to produce these antimicrobials and chemokines was investigated. Mouse gastroids prepared from wild-type C57BL/6 mice were stimulated for 6 h with recombinant IL-17A, IL-17F, or IL-17A/F, and real-time qPCR was performed to measure the relative expression of *Cxcl1*, *S100a8*, *S100a9*, *Nox1*, *Pigr*, and *Bdef14*. *Cxcl1* was included as a representative neutrophil-recruiting chemokine because in previous experiments with human gastric epithelial cell lines it was upregulated when cells were cultured with IL-17A and a stimulus (35). Furthermore, in previous mouse model studies, *Cxcl1* was expressed at significantly lower levels in *H. pylori*–infected *IL17ra^−/−^* mice compared with *H. pylori*–infected C57BL/6 control mice (32). IL-17A (and to a lesser extent IL-17A/F) induced expression of *Nox1*, *Pigr*, and *Cxcl1* (Fig. 2B). Expression of *S100a8*, *S100a9*, and *Bdef14* was not upregulated in gastroids in these assays. These data suggest that IL-17 signaling through IL-17RA is sufficient for direct activation of gene expression of *Nox1*, *Pigr*, and *Cxcl1* in epithelial cells without a costimulus.

### IL-17RA deficiency in gastric epithelial cells provides a model to address cell specific responses to *H. pylori* infection in vivo

**FIGURE 3.**
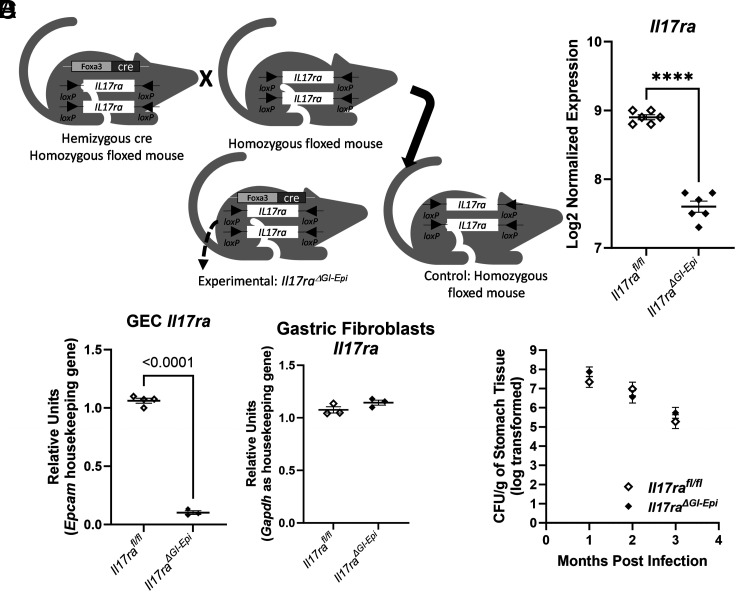
IL-17RA signaling in gastrointestinal epithelial cells is required for control of inflammation, not colonization of *H. pylori*. (**A**) Breeding scheme. (**B**) Differential expression of the *Il17ra* gene was determined using a multiplex RNA hybridization assay (Rosalind analysis) from RNA isolated from stomachs of *Il17ra^fl/fl^* versus *Il17ra^ΔGI-epi^* at 3 mo postinfection (also see Supplemental Table II). A *t* test was performed, and *p* value adjustment was performed using the Benjamini–Hochberg method of estimating false discovery rates. ****p < 0.0001. (**C**) Relative expression of *Il17ra* was determined in isolated gastric epithelial cells and isolated gastric fibroblasts using real-time RT-PCR comparing *Foxa3^cre^Il17ra^fl/fl^* mice (*Il17ra^ΔGI-Epi^*) to control mice. (**D**) CFU were determined across three different time points up to 3 mo postinfection by plating serial dilutions of stomach homogenates. The log of the CFU per gram of stomach tissue is presented. The number of mice in each group is between three and eight, and the CFU per gram are representative of one of two or three experiments at each time point.

To address the role of IL-17RA signaling in epithelial cells in vivo, the *Foxa3^cre^Il17ra^fl/fl^* strain was crossed with the *Il17ra^fl/fl^* strain (Fig. 3A). It was previously demonstrated that Foxa3-driven Cre recombinase activity occurs at embryonic day 8.5 in the anterior intestinal portal, and later in the entire gut endoderm from stomach to colon (39). Gene expression analysis using the multiplex RNA hybridization assay on RNA isolated from whole stomach tissue indicates that normalized expression of *Il17ra* is significantly reduced in *Foxa3^cre^Il17ra^fl/fl^* compared with controls, *Il17ra^fl/fl^* (Fig. 3B). Gene expression analysis of gastric epithelial cells from these mice and controls indicates that expression of the *Il17ra* gene was at the lower limit of detection in *Foxa3^cre+^* gastric epithelial cells compared with gastric epithelial cells from control mice (Fig. 3C). Furthermore, gastric fibroblasts from *Foxa3^cre^Il17ra^fl/fl^* mice retain expression of *Il17ra* (Fig. 3C). Other groups have also used the Foxa3cre model to investigate epithelial cell–specific responses, and although the nomenclature has not been consistent (40–44), we have chosen to use the nomenclature *Il17ra^ΔGI-Epi^* to describe these mice in this study. This allowed us to investigate the role of IL-17RA in epithelial cells during *H. pylori* infection in the stomach (see *Materials and Methods* assays used to validate the *Foxa3^cre^Il17ra^fl/fl^*).

At 2 mo of age, mice were challenged by orogastric gavage with two doses of *H. pylori* strain PMSS1. The course of infection was followed in both control (*Il17ra^fl/fl^* mice) and *Il17ra^ΔGI-epi^* mice (*Foxa3^cre^Il17ra^fl/fl^*) for up to 3 mo postinfection. Loss of IL-17RA expression in epithelial cells did not result in any change in bacterial burden after *H. pylori* infection for there was no significant difference in *H. pylori* colonization between control mice (*Il17ra^fl/fl^* mice) and *Il17ra^ΔGI-epi^* mice at 1, 2, or 3 mo postinfection (Fig. 3D).

### A deficiency in IL-17RA signaling in epithelial cells is sufficient to drive increased inflammation

**FIGURE 4.**
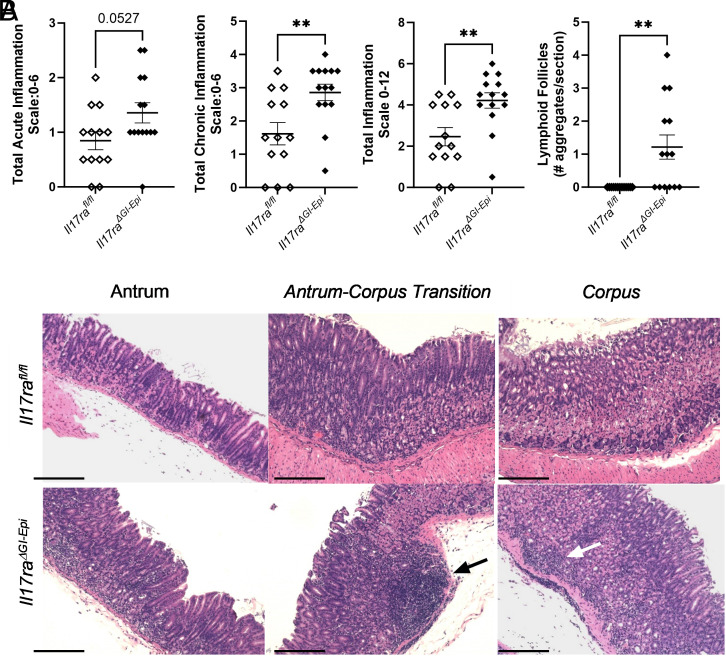
*Il17ra^ΔGI-epi^* mice display increased inflammation in response to *H. pylori* infection. (**A**) Acute and chronic inflammation was scored in stomach tissue at 3 mo after *H. pylori* infection by a blinded pathologist. Acute inflammation scores are based on the density of neutrophils, and chronic inflammation scores are based on the density of lamina propria mononuclear leukocyte infiltration. Lymphoid follicles and aggregates were also quantified per section. Error bars represented mean ± SEM. Statistical analysis was performed using a Mann–Whitney *U* test. **p < 0.01. (**B**) Representative H&E-stained antrum, antrum–corpus transition, and corpus tissue at original magnification of ×200 (scale bars, 200 µm). In the *Il17ra^ΔGI-epi^* panels, the black arrow points to an example lymphoid follicle, and the white arrow points to a representative lymphoid aggregate.

We previously observed that *Il17ra^−/−^* mice infected with *H. pylori* exhibited a reduction in acute inflammation (neutrophil infiltration) compared with wild-type mice (32). However, IL-17RA deficiency was also associated with an increase in chronic inflammation and often led to the development of lymphoid follicles in the gastric mucosa (32, 34), which was a rare event in wild-type mice (32, 33). Gastric inflammation was also scored in this study. However, acute inflammation scores are based on the density of neutrophils, and chronic inflammation scores are based on the density of lamina propria mononuclear leukocyte infiltration (mainly lymphocytes, plasma cells, and macrophages) within each sample. We find that by 3 mo postinfection the *Il17ra^ΔGI-epi^* mice have an increase in both acute and chronic inflammation compared with control mice (Fig. 4A). Furthermore, these *Il17ra^ΔGI-Epi^* mice are more likely to develop lymphoid follicles in their gastric mucosa as demonstrated by the number of lymphoid follicles or lymphocyte aggregates observed in H&E-stained sections (Fig. 4B, Supplemental Table II) where about half of the *Il17ra^ΔGI-Epi^* mice develop two to four lymphoid follicles or lymphoid aggregates per section and control mice do not develop these aggregates or follicles. The increased infiltration of immune cells in the *Il17ra^ΔGI-epi^* mice as compared with controls, occurs both in the antrum and the corpus regions of the stomach. However, the most pronounced infiltrate is at the corpus–antrum transitional zone (Fig. 4B). These data indicate that loss of *Il17ra* in epithelial cells of the gastrointestinal tract is sufficient to drive increased inflammation in the gastric mucosa in response to *H. pylori* infection.

### IL-17RA deficiency in the intestinal epithelial cells is not sufficient to drive increased inflammation in response to *H. pylori* infection

The experiments performed in the *Il17ra^ΔGI-Epi^* mice were designed to investigate the role of IL-17RA in the stomach and because Villin is not expressed in the stomach, the *Vil^cre^* model was not an option for our studies. At the same time, *Foxa3^cre^* models are known to impact intestinal epithelium (41, 42); therefore, our findings in the *Il17ra^ΔGI-Epi^* mice do not rule out a potential role for IL-17RA on intestinal epithelium and an impact on *H. pylori* immunopathogenesis. IL-17RA deficiency in the intestines has been shown to impact the microbiome (52, 53, 59), and the microbiome can impact homeostatic levels of Th17 cells (60–63). Therefore, we infected *Il17ra^ΔIEC^* (*Vil^cre^Il17ra^fl/fl^*) mice and littermate controls, *Il17ra^fl/fl^* mice, with *H. pylori* to determine whether the impact on Th17 responses and the microbiome might drive changes in *H. pylori* immunopathogenesis. By 3 mo postinfection, when differences in inflammation are observed in germline *Il17ra^−/−^* and *Il17ra^ΔGI-Epi^* mice compared with appropriate control mice (Fig. 4), there were no significant differences in bacterial burden or inflammation observed in *Il17ra^ΔIEC^* mice and littermate controls, *Il17ra^fl/fl^* (Supplemental Fig. 1). These data suggest that gastric epithelial cell responses at the site of *H. pylori* infection must be impacted directly by the loss of IL-17RA for the development of exacerbated inflammation.

### Genes encoding proteins engaged in lymphocyte trafficking, cell adhesion, IFN signaling, and lymphocyte activation are differentially regulated in *Il17ra^ΔGI-Epi^* mice

**FIGURE 5.**
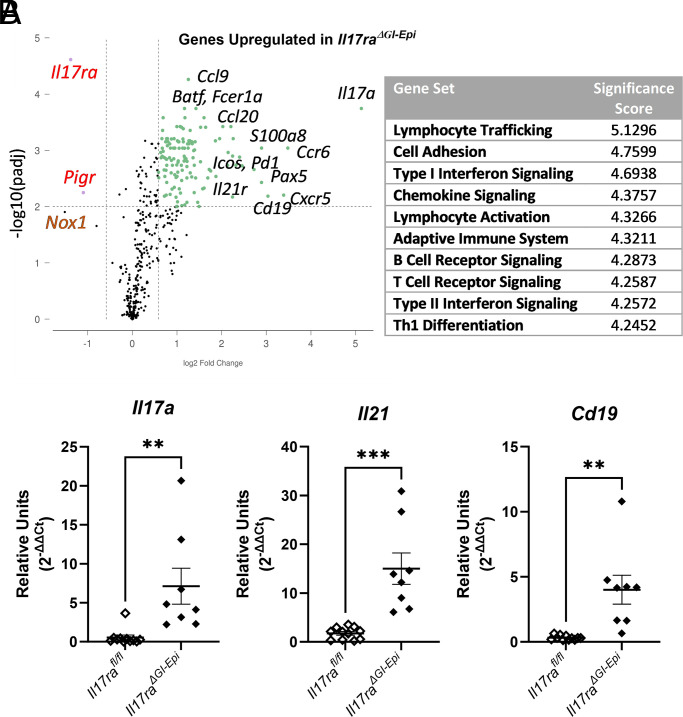
*H. pylori* infection of *Il17ra^ΔGI-epi^* mice results in the upregulation of proinflammatory genes. (**A**) Differential expression of a number of inflammatory genes was determined using the NanoString immunology panel (Rosalind analysis) from RNA isolated from stomachs of *Il17ra^fl/fl^* versus *Il17ra^ΔGI-Epi^* at 3 mo postinfection. Loss of the *Il17ra* in epithelial cells leads to enrichment of pathways involved in cell trafficking and T lymphocyte and B lymphocyte activation (complete listing of differentially expressed genes is in Supplemental Table I). In this panel, two genes were significantly less abundant in *Il17ra^ΔGI-Epi^* compared with controls, *Il17ra*, and *Pigr.* (**B**) Real-time qPCR was performed to confirm differential gene expression of key proinflammatory genes. Data are representative of three independent experiments; individual values from each sample are shown with the SEM for each group. An unpaired *t* test was performed to assess statistical significance. **p < 0.01, ***p < 0.001.

A multiplex RNA hybridization assay was performed to investigate how the gene expression patterns in gastric tissue of *Il17ra^ΔGI-Epi^* mice compared with controls, as well as to compare the differential expression analysis results from the germline *Il17ra^−/−^* and *C57BL/6* mice (Fig. 2A). RNA was isolated from gastric tissue at 3 mo postinfection, and the multiplex RNA hybridization assay was performed using NanoString’s mouse immunology panel. The data indicate that many genes were differentially regulated in the *Il17ra^ΔGI-Epi^* mice compared with control mice at this time point (volcano plot, Fig. 5A). Upregulated genes encode proteins engaged in cellular activities such as lymphocyte trafficking, cell adhesion, IFN signaling, and lymphocyte activation based on annotation by PANTHER. A complete list of the differentially regulated genes can be found in Supplemental Table III. It is notable that among the genes exhibiting a >1.5 log_2_ fold increase in the *Il17ra^ΔGI-Epi^* mice are those genes associated with Th17 and T follicular helper cell responses, including *Il17a*, *Ccr6*, *Cxcr5*, *Il21r*, and *Ccl20* (Supplemental Table III). To confirm that these responses were elevated the Th17/T follicular helper cytokine, *Il21*, and the Th17 cytokine, *Il17a*, were measured in gene expression analysis (Fig. 5B). Furthermore, it is known that IL-21 can drive B cell activation and isotype switching in lymphoid tissues. With evidence that B cell–associated genes, including *Cd19*, *Pax5*, and *Btla* are among the most highly upregulated in the *Il17ra^ΔGI-Epi^* mice, relative *Cd19* expression was also measured. These real-time qPCR analyses confirm that Th17 and B cell responses are increased in the *Il17ra^ΔGI-Epi^* stomach tissues with increased relative expression of *Cd19*, *Il17a*, and *Il21* (Fig. 5B).

### Epithelial cell response to IL-17 is not necessary to activate neutrophil recruitment

**FIGURE 6.**
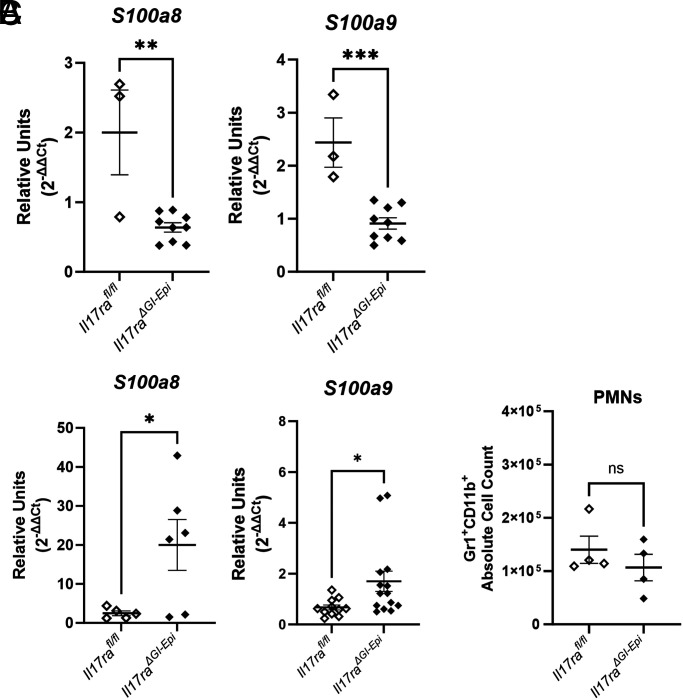
PMN recruitment to the stomach increases in *Il17ra^ΔGI-epi^* mice by 3 mo after *H. pylori* infection. (**A** and **B**) Levels of expression of neutrophil-expressing genes, including *S100a8* and *S100a9*, were assessed by real-time qPCR analysis of RNA isolated from stomachs of mice infected for (A) 1 mo and (B) 3 mo postinfection. Relative units are conveyed where relative units (2^−ΔΔCt^) refer to the sample calibration performed using an endogenous control gene (*Gapdh*) and then a calibrator sample (uninfected *Il17ra^fl/fl^* sample). Individual values from each sample are shown with the SEM for each group. An unpaired *t* test was performed to assess statistical significance. *p < 0.05, **p < 0.01, ***p < 0.001. (**C**) Flow cytometry was performed to enumerate neutrophils (PMNs, CD11b^+^Gr1^+^) in the gastric tissue. An unpaired *t* test was performed to assess statistical significance.

In many models of bacterial or fungal infection, including *H. pylori* infection, in germline IL-17RA–deficient mice, neutrophil recruitment was impaired (32, 54, 55, 58). In this study among the genes, which are significantly differentially regulated in the quantification of gene transcripts, is the *S100a8* gene. S100a8 and S100a9 proteins heterodimerize to form calprotectin, an antimicrobial protein (64). This protein is highly abundant in the cytoplasm of neutrophils (65–67), and therefore the expression of the genes and/or proteins for S100a8 or S100a9 serves as an excellent marker for neutrophil infiltration. Consistent with the finding that the *Il17ra^ΔGI-Epi^* mice develop acute inflammation after *H. pylori* infection (Fig. 4A), mice of this genotype also express higher levels of the *S100a8* and *S100a9* gene compared with infected control mice (Supplemental Table III). To investigate whether epithelial responses to IL-17 might be necessary for neutrophil infiltration early, the expression of *S100a8* and *S100a9* was also measured by real-time qPCR (Fig. 6). The relative expression of the *S100* genes is lower in the *Il17ra^ΔGI-Epi^* mice compared with control mice at the early time point (1 mo postinfection, Fig. 6A), but higher in the *Il17ra^ΔGI-Epi^* mice compared with control mice at the later time point (3 mo postinfection, Fig. 6B, Supplemental Table III). Flow cytometry was preformed to quantify neutrophils in the gastric tissues at 3 mo postinfection, and there is evidence that the *Il17ra^ΔGI-Epi^* mice and control mice do have similar numbers of PMNs infiltrating their tissues (Fig. 6C). These data suggest that the loss of IL-17RA in epithelial cells does not impact long-term neutrophil recruitment and that IL-17 may signal through other cell types to activated chemokine expression and neutrophil recruitment.

### IL-17 signaling in epithelial cells contributes to *Pigr* expression in mice, but gastric IgA levels are driven by chronic inflammation even with reduced *Pigr* levels

**FIGURE 7.**
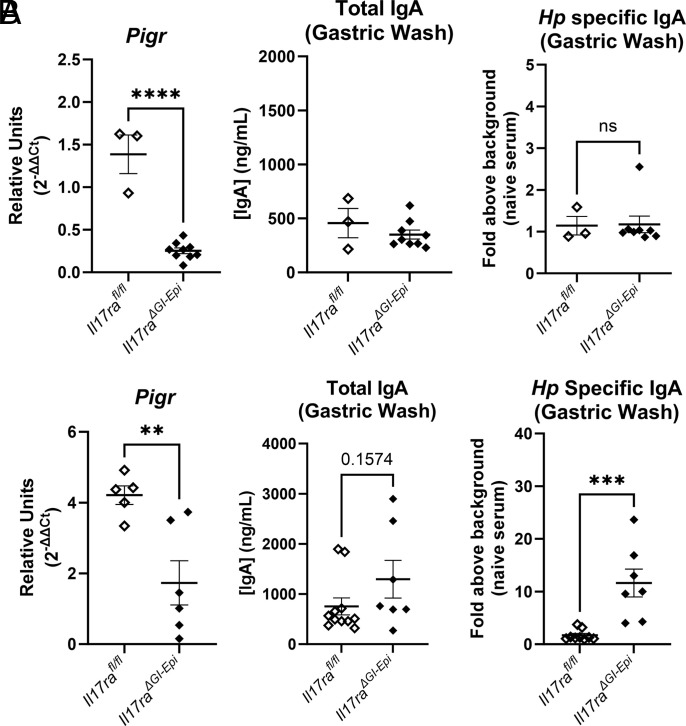
Despite reduced expression of *Pigr* in *Il17ra^ΔGI-epi^* mice after *H. pylori* infection, IgA levels increase in the gastric mucosa. (**A** and **B**) Real-time qPCR analysis of *Pigr* expression was performed on RNA isolated from gastric tissue, and levels of IgA were determined by ELISA at (A) 1 mo postinfection and (B) 3 mo postinfection. Data are representative a minimum of two experiments. Real-time qPCR data are expressed in relative units using *Gapdh* as the housekeeping gene and uninfected gastric RNA as the calibrator sample. Individual values from each sample are shown with the SEM for each group. An unpaired *t* test was performed to assess statistical significance. **p < 0.01, ***p < 0.001, ****p < 0.0001.

The polymeric Ig receptor (pIgR) has been shown to be regulated by IFN-γ in several tissues, including lung and intestines, and by IL-17 in the intestines (50). The role of IgA response to controlling microbial colonization and inflammation in the intestines is clear (68–70), but the role of pIgR and IgA in the stomach is not well understood. The multiplex RNA hybridization assay demonstrated that the abundance of *Pigr* transcripts is reduced in *H. pylori*–infected *Il17ra^−/−^* and *Il17ra^ΔGI-Epi^* mice at 3 mo postinfection compared with C57BL/6 mice or *Il17ra^fl/fl^* mice, respectively (Fig. 2A, Supplemental Tables I, III). Real-time qPCR was performed to address whether this was consistent at other timepoints. At both timepoints, 1 and 3 mo postinfection, there is significantly lower expression of *Pigr* in the gastric tissue of *Il17ra^ΔGI-Epi^* mice compared with control mice (Fig. 7). These data are calibrated to uninfected *Il17ra^fl/fl^* stomach tissue. To investigate whether this reduced expression of *Pigr* also resulted in an IgA deficiency in the gastric lumen, ELISAs were performed to measure both total IgA and *H. pylori*–specific IgA levels. Despite this, there is no significant difference in IgA levels in the gastric wash; in fact, by 3 mo postinfection there is a significant increase in *H. pylori*–specific IgA levels in the *Il17ra^ΔGI-Epi^* mice (Fig. 7B). It is surprising that high levels of IgA are still observed in the gastric wash with lower levels of *Pigr.* There are at least two possible explanations. One explanation is that *Pigr* is not absent, but is expressed at a significantly lower level; therefore, there may be sufficient *Pigr* expression for translocation of IgA. The other plausible explanation is that the barrier function is not fully maintained in the absence of IL-17RA signaling in epithelial cells and IgA might leak into the luminal space. Either way, the observation of increased IgA levels in the *Il17ra^ΔGI-epi^* mice suggests that there could be increased Ag stimulation leading to chronic inflammation in the absence of IL-17RA expression in epithelial cells.

### Primary gastric fibroblasts respond to IL-17A producing neutrophil recruiting chemokines

**FIGURE 8.**
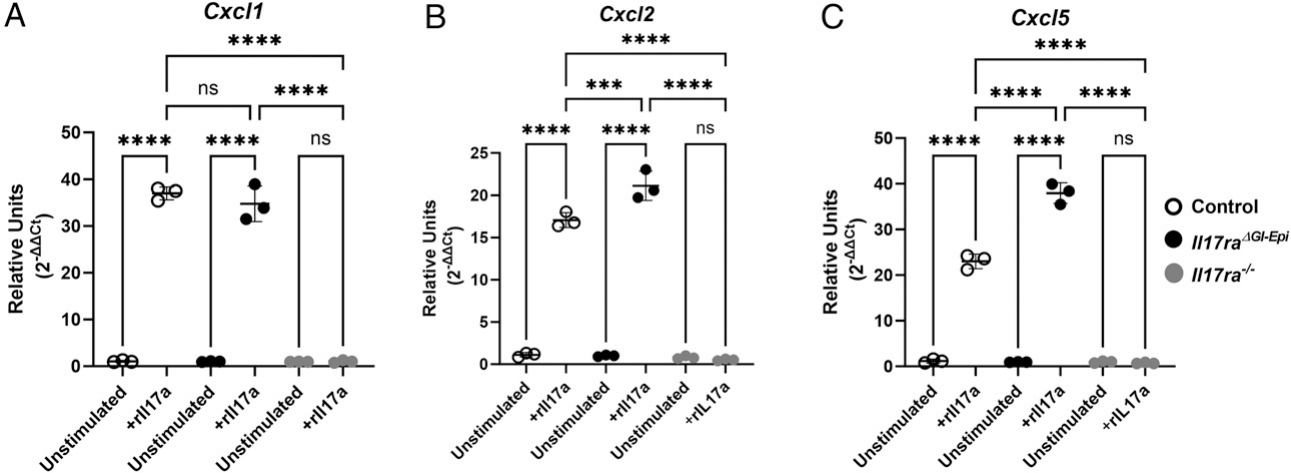
Fibroblasts respond to IL-17, expressing PMN recruiting chemokines. (**A–C**) Primary fibroblasts were cultured from uninfected gastric tissue from C57BL/6, *Il17ra^ΔGI-Epi^*, and germline *Il17ra^−/−^* mice, expanded in culture, and then stimulated with 100 ng/ml rIL-17a for 5 h. Real-time RT-PCR was performed to measure relative expression of PMN recruiting chemokines, (A) *Cxcl1*, (B) *Cxcl2*, and (C) *Cxcl5*. Data are expressed in relative units using *Gapdh* as the housekeeping gene and unstimulated fibroblasts as the calibrator sample. Individual values from each sample are shown with the SD for each group. One-way ANOVA was performed with a Dunnett multiple comparison test to assess statistical significance compared with unstimulated cells. ***p < 0.001, ****p < 0.0001.

The data presented thus far indicate that *Il17ra^ΔGI-Epi^* mice are capable of recruiting PMNs during chronic infection in response to *H. pylori* infection. This is in contrast to both published results and those results in this current study, which indicate that *H. pylori*–infected germline *Il17ra^−/−^* mice lack IL-17RA on all cell types and have a PMN deficiency compared with *H. pylori*–infected C57BL/6 controls (32, 33). To determine whether fibroblasts in the gastric tissue could be responsible for recruiting PMNs, primary gastric fibroblasts were extracted and cultured from the stomach and then stimulated with IL-17a (Fig. 8). The fibroblasts respond to IL-17a by upregulating expression of PMN recruiting chemokines including *Cxcl1*, *Cxcl2*, and *Cxcl5.* To confirm that fibroblasts isolated from *Il17ra^ΔGI-Epi^* mice retain IL-17RA and can respond to IL-17a, the relative expression of *Cxcl1*, *Cxcl2*, and *Cxcl5* was compared in unstimulated and IL-17a–stimulated fibroblasts from C57BL/6, *Il17ra^ΔGI-Epi^*, and germline *Il17ra^−/−^* mice by real-time RT-PCR. Fibroblasts from *Il17ra^ΔGI-Epi^* mice responded similarly to recombinant IL-17a as for C57BL/6 mice, and, as expected, germline *Il17ra^−/−^* fibroblasts did not respond to the stimulation (Fig. 8). These data indicate that fibroblasts could contribute to PMN recruitment during infection and may be responsible in this model for IL-17–mediated infiltration of PMNs.

## Discussion

The exacerbated inflammatory response during chronic *H. pylori* infection was originally described several years ago, but how IL-17RA mediates a protective role in limiting inflammation has been difficult to elucidate because so many cell types express IL-17RA. One hypothesis we explored previously in the *H. pylori* infection model was that IL-17RA signaling in T cells was facilitating a negative feedback loop, and this negative feedback was necessary to downregulate the inflammatory Th17 cells. It was previously demonstrated that T cell–intrinsic IL-17 acts in an autocrine negative feedback loop (71). We saw evidence of this, even in the absence of *H. pylori* infection, where *Il17ra^−/−^* mice expressed 2- to 3-fold more *Il17a* than do naive C57BL/6 controls (32). More recently, this inhibitory activity of IL-17A was explored in an autoimmne uveitis model (EAU) (72). Chong et al. (72) demonstrated that loss of IL-17A signaling in autopathic Th17 cells led to increased expression of Th17-associated cytokines. Their data suggest that IL-17A signaling via IL-17RA results in elevated expression of IL-24. IL-24, in turn, acts on suppressors of cytokine synthesis (SOCS) 1 and 3, which typically leads to downregulation of GM-CSF and IL-17. To address this in our model of *H. pylori* infection in mice, we used the *Cd4^cre^Il17ra^fl/fl^* mice to create T cell–specific-IL-17RA–deficient mice. The findings demonstrated that loss of IL-17RA in T cells alone was not sufficient for *H. pylori*–induced hyperinflammation (33) leading us to move on to the additional hypothesis that epithelial cell defects in IL-17RA signaling could impact epithelial cell barrier integrity during *H. pylori* infection.

In the current study, we found that loss of IL-17RA in epithelial cells is sufficient for the development of *H. pylori*–induced hyperinflammatory responses (relative to control mice). The data in the current study suggest that the loss of IL-17RA in gastrointestinal epithelial cells alone is sufficient to drive increased expression of Th17 cytokines, including *Il21* and *IL17a*. Several genes that are linked to barrier integrity are also impacted by the loss of IL-17RA expression, including *Nox1*, *Pigr*, and *Cxcl1.* Interestingly, in the in vivo model, as time progresses and the augmented expression of T cell cytokines becomes more pronounced, significant trends emerge concerning inflammation. First, there is a resulting increase in B cell activation, and lymphoid follicles form in the gastric tissue of many of the *H. pylori*–infected IL-17RA^Δepi^ mice, which is not a pathological outcome in control mice. Second, by 3 mo postinfection, when levels of IL-17A are significantly increased in the *H. pylori*–infected *Il17ra^ΔGI-Epi^* mice, there is an influx of neutrophils that was never observed in the *H. pylori*–infected germline *Il17ra^−/−^* mice. This finding was supported by histological scoring (acute inflammation score), flow cytometry, and real-time qPCR (S100 gene expression). The neutrophil infiltration in the *Il17ra^ΔGI-Epi^* mice suggests that IL-17 mediates neutrophil recruitment through a cell type different from epithelial cells in the stomach. Although gastric epithelial cells do respond to IL-17A by producing neutrophil recruiting chemokine *Cxcl1*, the results presented in this study demonstrate that primary gastric fibroblasts also respond to IL-17A. Fibroblasts upregulated chemokines known to activate neutrophil migration including *Cxcl1*, *Cxcl2*, and *Cxcl5* in response to IL-17A.

The phenotype characterized by exacerbated inflammation and Th17 responses following *H. pylori* infection, which was observed in IL-17RA^ΔGI-epi^ mice, is not observed in the IL-17RA^ΔIEC^ (*Vil^cre^Il17ra^fl/fl^*) mice. This suggests that IL-17RA has a role specifically in the stomach epithelium and as a protective activation pathway against immunopathological consequence of *H. pylori* infection. Altered microbiome and lower mucosal IgA levels that have been reported in the intestines due to IL-17RA deficiency by others (50, 52, 59) are likely not driving the increased inflammation in the stomach during *H. pylori* infection of the IL-17RA^Δepi^ mice compared with controls. The impact of loss of IL-17RA needs to be at the site of *H. pylori* infection for exacerbated inflammation to occur. The role of the IgA response in control of microbial colonization and inflammation in the intestines is clear (61–63), but the role of pIgR and IgA in the stomach is not well understood. Our findings suggest that IL-17a can regulate *Pigr* expression in epithelial cells, but, furthermore, that IgA levels in the stomach are not always dictated by the level of *Pigr.* In this model of IL-17RA deficiency, the exacerbated inflammatory response is characterized by increased B cell infiltration and a high level of IgA production. This is interesting, as it is in contrast to what was observed in intestinal models. In a previous study using *Pigr^−/−^* mouse (73), the data suggested a mild impact on *H. pylori* colonization in the duodenum (which was not a consistent finding) at time points beyond 6 mo. Unfortunately, the study did not address immunopathological consequences in the tissues. Taken together, the data suggest that IgA is not contributing to control or clearance of *H. pylori* infection in the stomach, but is either a marker of exacerbated immune activation or could be contributing to it.

A head-to-head comparison of *H. pylori* infection and chronic inflammation between *Il17ra^−/−^* mice, *Il17ra^ΔGI-Epi^* mice, and their respective controls was not performed. These mice are not on identical genetic backgrounds and are bred in different housing rooms, so it would not have been a perfectly controlled experiment. Comparing data from experiments performed in the same biosafety level-2 facility, within weeks of each other and with the same strains of *H. pylori*, it does appear that a greater percentage of *Il17ra^−/−^* mice develop lymphoid follicles by 3 mo postinfection compared with the IL-17RA^ΔGi-epi^ mice. This suggests that while loss of IL-17RA signaling in epithelial cells is sufficient to drive increased inflammation and lymphoid follicle development, IL-17 signaling in other cell types is also playing a role in modulating inflammation. It may be that the fibroblast response to IL-17 and the recruitment of neutrophils actually reduces antigenic load and/or influences the barrier maintenance. A limitation of this model is that expression of *Foxa3* has been described in tissues outside of the stomach, including pancreas, testes, and liver. Therefore, there may be some cells in these tissues that are also impacted by the *Foxa3^cre^* conditional deletion of *Il17ra.* It is unlikely that IL-17RA expression in these tissues impacts immunopathogenesis in the stomach, but we cannot rule out this possibility entirely.

The in vivo and in vitro data presented in this study provide evidence that IL-17R signaling though epithelial cells is a vital component of the host response to *H. pylori* infection.

## Supplementary Material

Supplemental Material (PDF)
