# Supplementary material for: IL-17RA–Mediated Epithelial Cell Activity Prevents Severe Inflammatory Response to Helicobacter pylori Infection
Source: Immunohorizons. 2024 Apr 19;8(4):339–53. doi: 10.4049/immunohorizons.2300078 (PMC11066722; doi:10.4049/immunohorizons.2300078)
Supplement: Supplemental Material (PDF) [file IH_2300078_Supplemental_1.pdf]

Supplemental Figure 1. *IL-17A signaling in intestinal epithelial cells (Villin expressing cells) is not required for control of stomach inflammation or colonization of H. pylori*. (A) CFU were determined at 3 months post infection by plating serial dilutions of stomach homogenates. The Log of the CFU per gram of stomach tissue is presented (a t-test was performed on the data). (B) Hematoxylin and eosin-stained sections were used to score acute and chronic inflammation in the stomach at 3 months post-*H. pylori* infection and total inflammation was calculated (Scale 0-12), Mann-Whitney U test was performed on the data (no significant differences). These data are representative of 3 independent experiments with n=6-9 per group in each independent experiment.

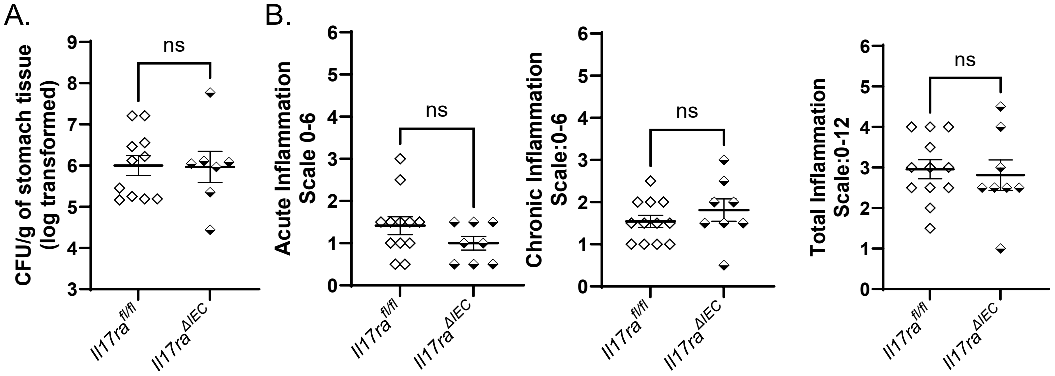

Supplemental Table I. Differential Expression Analysis Nanostring Immunology Panel of *Il17ra*$^{-/-}$ mice and *C57Bl/6* controls at 3 mpi. All genes which had differential abundance are presented in the table if they had > or < 1.5 Log2Fold Change and a P-value of <0.01.

Gene Symbol	**Description**	**Log2Fold Change**	**P-Value**	**P-Adj**
Cd19	CD19 antigen	4.58397	6.25E-06	0.000131
Cxcr5	chemokine (C-X-C motif) receptor 5	4.18488	3.38E-05	0.000345
Ms4a1	membrane-spanning 4-domains, subfamily A, member 1	4.15761	3.23E-07	4.14E-05
Pax5	paired box 5	4.02647	4.50E-07	4.14E-05
Il21r	interleukin 21 receptor	3.84235	0.000219	0.001258
Tnfrsf13c	TNF receptor superfamily, member 13c	3.23266	0.000705	0.002794
Cd79b	CD79B antigen	3.21072	3.82E-07	4.14E-05
H2-ob	histocompatibility 2, O region beta locus	3.18322	1.65E-06	9.96E-05
Ccr6	chemokine (C-C motif) receptor 6	3.08438	7.42E-06	0.000131
Cd22	CD22 antigen	3.01314	0.000104	0.000725
Il17a	interleukin 17A	2.81889	1.45E-05	0.000209
Btnl1	butyrophilin-like 1	2.75132	0.000272	0.001487
Btla	B and T lymphocyte associated	2.68187	1.20E-05	0.000191
Ccl19	chemokine (C-C motif) ligand 19	2.58339	2.37E-06	9.96E-05
Ctla4	cytotoxic T-lymphocyte-associated protein 4	2.48755	1.37E-05	0.000203
Slamf1	signaling lymphocytic activation molecule family member 1	2.4468	6.65E-06	0.000131
H2-dmb2	histocompatibility 2, class II, locus Mb2	2.41727	0.000507	0.002201
Tigit	T cell immunoreceptor with Ig and ITIM domains	2.40269	1.65E-05	0.000229
Ikzf3	IKAROS family zinc finger 3	2.38427	5.15E-05	0.000483
Cxcl11	chemokine (C-X-C motif) ligand 11	2.3779	0.003925	0.01062
Sell	selectin, lymphocyte	2.2204	0.000147	0.000916
Icos	inducible T cell co-stimulator	2.1775	4.55E-05	0.000436
Sh2d1A	SH2 domain containing 1A	2.13578	0.000406	0.001889
Map4k1	mitogen-activated protein kinase kinase kinase kinase 1	2.10692	2.97E-05	0.000318
Btnl2	butyrophilin-like 2	2.09032	0.00037	0.001795
Stat4	signal transducer and activator of transcription 4	2.08037	0.000463	0.002089
Cxcl13	chemokine (C-X-C motif) ligand 13	2.02872	6.56E-06	0.000131
Ikzf1	IKAROS family zinc finger 1	2.02056	2.38E-06	9.96E-05
Cd2	CD2 antigen	1.99225	7.34E-06	0.000131
Ccl20	chemokine (C-C motif) ligand 20	1.97461	2.95E-05	0.000318
Cd27	CD27 antigen	1.90847	7.55E-05	0.00056
Ifit2	interferon-induced protein with tetratricopeptide repeats 2	1.87762	0.000355	0.001754
Cd53	CD53 antigen	1.85946	7.87E-06	0.000134
Pdcd1	programmed cell death 1	1.85283	2.62E-05	0.000318
Stat2	signal transducer and activator of transcription 2	1.8481	0.000318	0.001609
Spn	sialophorin	1.83506	6.02E-06	0.000131
Fcamr	Fc receptor, IgA, IgM, high affinity	1.83335	0.000308	0.001581
Cd247	CD247 antigen	1.80837	0.000117	0.000777
Cxcl10	chemokine (C-X-C motif) ligand 10	1.80655	0.009655	0.022415
Tnfsf10	tumor necrosis factor (ligand) superfamily, member 10	1.79063	0.000127	0.000824
Pou2f2	POU domain, class 2, transcription factor 2	1.78143	3.85E-06	0.000104
Il27ra	interleukin 27 receptor, alpha	1.78069	0.000479	0.002137
Lef1	lymphoid enhancer binding factor 1	1.77879	0.000721	0.002835
Btk	Bruton agammaglobulinemia tyrosine kinase	1.77647	9.02E-06	0.000148
Ltb	lymphotoxin B	1.76345	0.000461	0.002089
Ccl5	chemokine (C-C motif) ligand 5	1.74419	0.007476	0.0181
Cd3E	CD3 antigen, epsilon polypeptide	1.67635	0.000705	0.002794
Lck	lymphocyte protein tyrosine kinase	1.66961	0.000286	0.001548
Cd6	CD6 antigen	1.65344	0.000371	0.001795
Il12rb1	interleukin 12 receptor, beta 1	1.64712	0.001294	0.004375
Il21	interleukin 21	1.63743	0.002669	0.007723
Zap70	zeta-chain (TCR) associated protein kinase	1.61566	0.001556	0.005078
Traf1	TNF receptor-associated factor 1	1.60779	0.002944	0.008422
Itga4	integrin alpha 4	1.59295	3.56E-06	0.000104
Il16	interleukin 16	1.59275	5.80E-06	0.000131
Irf7	interferon regulatory factor 7	1.58415	0.001126	0.003954
Cd3D	CD3 antigen, delta polypeptide	1.57784	0.006812	0.017123
Ptprc	protein tyrosine phosphatase, receptor type, C	1.57324	1.80E-05	0.000237
Ciita	class II transactivator	1.57183	0.003355	0.009298
Il17Ra	interleukin 17 receptor A	1.57026	1.88E-08	8.64E-06
Ptpn22	protein tyrosine phosphatase, non-receptor type 22 (lymphoid)	1.56403	5.60E-05	0.000515
Cd40	CD40 antigen	1.54977	0.00021	0.001222
Irf4	interferon regulatory factor 4	1.5193	0.000161	0.000985
Gene Symbol	**Description**	**Log2 fold**	**P-Value**	**P-Adj**
S100a8	S100 calcium binding protein A8 (calgranulin A)	-4.40338	2.15E-06	9.96E-05
Cxcl15	chemokine (C-X-C motif) ligand 15	-4.12763	2.87E-05	0.000318
Nox1	NADPH oxidase 1	-4.07967	2.85E-05	0.000318
S100a9	S100 calcium binding protein A9 (calgranulin B)	-2.61837	6.82E-05	0.000541
Pigr	polymeric immunoglobulin receptor	-2.37092	3.40E-06	0.000104
Cxcr2	chemokine (C-X-C motif) receptor 2	-2.13848	0.000985	0.003568
Itln1	intelectin 1 (galactofuranose binding)	-2.05201	3.70E-06	0.000104
Defb14	defensin beta 14	-1.43945	0.001619	0.005172

Supplemental Table II. Percentage of *H. pylori* infected mice with gastric lymphoid follicles or lymphoid aggregates by 3 months post infection. Lymphoid follicles and aggregates were quantified by the pathologist per section (representative in Fig 4A). The % of animals that had lymphoid follicles or aggregates are defined below in 3 independent experiments per genotype.

Experiment (3 mo P.I.)	*Experimental Group*	*Control Group*
$Il17ra^{-/-}$ vs C57Bl/6 (Exp1)	89% (8/9)	0% (0/7)
$Il17ra^{-/-}$ vs C57Bl/6 (Exp 2)	88% (7/8)	0% (0/9)
$Il17ra^{-/-}$ vs C57Bl/6 (Exp 3)	57% (4/7)	0% (0/6)
$Il17ra^{\Delta GI\text{-}Epi}$ vs $Il17ra^{fl/fl}$ (Exp 1)	43% (3/7)	0% (0/7)
$Il17ra^{\Delta GI\text{-}Epi}$ vs $Il17ra^{fl/fl}$(Exp 2)	57% (4/7)	0% (0/10)
$Il17ra^{\Delta GI\text{-}Epi}$ vs $Il17ra^{fl/fl}$(Exp 3)	57% (8/14)	0% (0/13)

Supplemental Table III. Differential Expression Analysis Nanostring Immunology Panel at 3 mpi in *IL17ra*$^{\Delta GI\text{-}Epi}$ mice to *Il17ra*$^{fl/fl}$ controls. All genes which had differential abundance are presented in the table if they had > or < 1.25 Log2Fold Change and a P-value of <0.01.

Gene	**Description**	**Log2Fold Change**	**P-Value**	**P-Adj**
Il17a	interleukin 17A	5.129283	1.39E-06	0.00018
Ccr6	chemokine (C-C motif) receptor 6	3.481779	0.000104	0.00091
Cxcr5	chemokine (C-X-C motif) receptor 5	3.388496	0.002108	0.006244
Cd19	CD19 antigen	3.036637	0.00227	0.006516
S100a8	S100 calcium binding protein A8 (calgranulin A)	2.893497	0.000102	0.00091
Pax5	paired box 5	2.893085	0.001085	0.003714
Ms4a1	membrane-spanning 4 _ subfamily A_ (encodes CD20)	2.725427	0.000495	0.002183
Cd22	CD22 antigen	2.457155	0.000406	0.00188
Sh2d1a	SH2 domain containing 1A	2.401448	0.000201	0.001316
H2-Ob	histocompatibility2-O region beta locus	2.31778	0.000393	0.001841
Sell	selectin lymphocyte	2.258626	3.34E-05	0.000619
Slamf1	signaling lymphocytic activation molecule family 1	2.245112	0.000184	0.001262
Il21r	interleukin 21 receptor	2.243346	0.002391	0.006734
Cxcl11	chemokine (C-X-C motif) ligand 11	2.206451	9.10E-06	0.000378
Btla	B and T lymphocyte associated	2.145127	0.000154	0.001089
Cd79b	CD79B antigen	2.132888	0.000393	0.001841
Ctla4	cytotoxic T-lymphocyte-associated protein 4	2.025868	1.02E-05	0.000378
Pdcd1	programmed cell death 1	1.954608	3.03E-05	0.000619
H2-Dmb2	histocompatibility 2 class II locus Mb2	1.87176	0.00079	0.002906
Icos	inducible T cell co-stimulator	1.860825	4.47E-05	0.000676
Ikzf3	IKAROS family zinc finger 3	1.75393	0.000563	0.002321
Cxcl13	chemokine (C-X-C motif) ligand 13	1.720375	0.000494	0.002183
Tnfsf11	tumor necrosis factor (ligand) superfamily_ 11	1.689299	0.000868	0.003088
Tigit	T cell immunoreceptor with Ig and ITIM domains	1.641207	1.22E-05	0.000388
Ccl19	chemokine (C-C motif) ligand 19	1.607261	0.001422	0.004688
Ccl20	chemokine (C-C motif) ligand 20	1.605008	4.72E-06	0.000262
Ccr3	chemokine (C-C motif) receptor 3	1.574694	0.001486	0.004826
Ccr7	chemokine (C-C motif) receptor 7	1.529091	1.31E-05	0.000388
S100a9	S100 calcium binding protein A9 (calgranulin B)	1.525699	0.000308	0.001594
Stat4	signal transducer and activator of transcription 4	1.494109	0.00395	0.009944
Klrc1	killer cell lectin-like receptor subfamily C 1	1.464108	0.003173	0.008384
Fcer1a	Fc receptor_ IgE_ high affinity I_ alpha	1.423928	1.76E-06	0.00018
Zap70	zeta-chain (TCR) associated protein kinase	1.417668	0.000882	0.003092
Cd2	CD2 antigen	1.40566	5.67E-05	0.000703
Ltb	lymphotoxin B	1.393145	0.000132	0.000983
Cxcl10	chemokine (C-X-C motif) ligand 10	1.387491	0.000383	0.001841
Lta	lymphotoxin A	1.384918	0.001824	0.005636
Cd40	CD40 antigen	1.372875	0.000138	0.00101
Irf7	interferon regulatory factor 7	1.359174	0.000225	0.001352
Btk	Bruton agammaglobulinemia tyrosine kinase	1.341737	7.01E-05	0.000761
Bst2	bone marrow stromal cell antigen 2	1.333275	5.58E-05	0.000703
Cd3d	CD3 antigen_ delta polypeptide	1.329618	0.000813	0.002964
Cd53	CD53 antigen	1.296393	0.000632	0.002512
Traf1	TNF receptor-associated factor 1	1.294655	1.77E-05	0.000442
Il27ra	interleukin 27 receptor_ alpha	1.288314	4.60E-05	0.000676
Itga2b	integrin alpha 2b	1.267933	7.18E-05	0.000761
Cxcr4	chemokine (C-X-C motif) receptor 4	1.265315	4.57E-05	0.000676
Ccl9	chemokine (C-C motif) ligand 9	1.25397	2.46E-07	5.48E-05
Lower Expression is below. Note: Nox1 expression was not <0.01 after P-Adj.				
Pigr	polymeric immunoglobulin receptor	-1.0977	0.00181	0.005632
Il17Ra	interleukin 17 receptor A	-1.38173	5.46E-08	2.43E-05
Nox1	NADPH oxidase 1	-1.51322	0.005186	0.012475
